# Mycolic Acid Biosynthesis Genes in the Genome Sequence of *Corynebacterium atypicum* DSM 44849

**DOI:** 10.1128/genomeA.00845-14

**Published:** 2014-08-21

**Authors:** Anna Tippelt, Sabrina Möllmann, Andreas Albersmeier, Sebastian Jaenicke, Christian Rückert, Andreas Tauch

**Affiliations:** a Institut für Genomforschung und Systembiologie, Centrum für Biotechnologie (CeBiTec), Universität Bielefeld, Bielefeld, Germany; b Bioinformatics Resource Facility, Centrum für Biotechnologie (CeBiTec), Universität Bielefeld, Bielefeld, Germany

## Abstract

The complete chromosomal sequence of the type strain *Corynebacterium atypicum* DSM 44849 comprises 2,311,380 bp. A functional annotation revealed the presence of genes involved in the synthesis and export of mycolic acids and in trehalose corynomycolate biosynthesis, supporting the view that the cell envelope of *C. atypicum* contains mycolic acids.

## GENOME ANNOUNCEMENT

The cell envelope of corynebacteria typically includes an outer layer of mycolic acids, which is functionally equivalent to the outer membrane of Gram-negative bacteria (1). However, a few species lack this component of the cell envelope, such as *Corynebacterium amycolatum* (2), *Corynebacterium caspium* (3), *Corynebacterium ciconiae* (4), *Corynebacterium lactis* (5), and *Corynebacterium kroppenstedtii* (6). The annotation of the complete genome sequence of *C. kroppenstedtii* DSM 44385 revealed that gene loss is responsible for the lack of mycolic acids in this species (7). Moreover, *Corynebacterium atypicum* originating from an unknown clinical source was found to lack the characteristic mycolic acids (8). On the contrary, a recent study demonstrated the presence of mycolic acids in the cell envelope of *C. atypicum* (5). We therefore determined the complete genome sequence of *C. atypicum* DSM 44849 to provide evidence of the presence of genes involved in mycolic acid biosynthesis.

Genomic DNA of the type strain *C. atypicum* DSM 44849 (initially named R2070) was obtained from the Leibniz Institute DSMZ (Braunschweig) and used to prepare a sequencing library with the Nextera DNA sample preparation kit (Illumina). The library was sequenced in a 2 × 300 nucleotide run using the MiSeq reagent kit version 3 and the MiSeq desktop sequencer (Illumina), resulting in 993,901 paired reads and 169,108,577 detected bases. The paired reads were assembled with the Roche GS *de novo* Assembler software (release 2.8) to yield 8 contigs in 5 scaffolds. The ordering of scaffolds was supported by the software r2cat (9), and the remaining gaps in the genome sequence were closed *in silico* with the Consed software (version 24) (10).

The genome sequence of *C. atypicum* DSM 44849 includes a circular chromosome of 2,311,380 bp, with a G+C content of 65.51%, and the circular corynephage ΦCATYP2070I, with a genome size of 48,068 bp and 58.18% G+C content. The annotation of the genome sequence was performed with the NCBI Prokaryotic Genome Annotation Pipeline and the GeneMarkS+ software (version 2.6) and was visualized with GenDB (version 2.2) (11). The annotation of the complete genome sequence revealed 1,578 protein-coding regions, 122 pseudogenes, 52 tRNA genes, 1 noncoding RNA gene, and 4 rRNA operons in *C. atypicum* DSM 44849.

The chromosome of *C. atypicum* DSM 44849 contains homologs of genes with proven functions in the biosynthesis and export of mycolic acids and in their transfer to the cell envelope. These genes encode the envelope lipid regulation factor ElrF (12), the conserved acyl-AMP ligase FadD1 (13), a unique condensase that performs the final condensation step of mycolic acid biosynthesis (13, 14), the conserved acyl-coenzyme A (CoA) carboxylase subunits AccD2 and AccD3 (13, 14), the carboxylase subunits AccBC and AccE (15), the Corynebacterineae mycolate reductase A (16), two membrane proteins of the MmpL family, which is involved in mycolic acid transport (17), and three mycolyltransferases (18, 19). This gene repertoire is consistent with the detection of mycolic acids in the cell envelope of *C. atypicum* DSM 44849 by thin-layer chromatography (6). It is therefore likely that *C. atypicum* is not an atypical (mycolic acid-free) corynebacterium.

### Nucleotide sequence accession numbers.

This genome project has been deposited in the GenBank database under accession numbers CP008944 (chromosome) and CP008945 (ΦCATYP2070I).
